# Network-Based Deep Brain Stimulation Rescue After Magnetic Resonance-Guided Focused Ultrasound Thalamotomy for Essential Tremor: Two Targeting Strategies

**DOI:** 10.5334/tohm.1279

**Published:** 2026-11-03

**Authors:** Francisco Aranda Godoy, Evan Wilson, Danielle Pietramalla, Christopher R. Honey, Stefan Lang

**Affiliations:** 1 Division of Neurosurgery, Department of Surgery, University of British Columbia, Vancouver, British Columbia, Canada; 2 Department of Neurosurgery, Pontifical Catholic University of Chile, Santiago, Chile

**Keywords:** essential tremor, magnetic resonance-guided focused ultrasound, deep brain stimulation, posterior subthalamic area, rubral wing, dentato-rubro-thalamic tract

## Abstract

**Background:**

Tremor may recur after magnetic resonance-guided focused ultrasound (MRgFUS) thalamotomy, and the optimal target for deep brain stimulation (DBS) rescue remains uncertain.

**Cases:**

A 71-year-old man with recurrent essential tremor after ventral intermediate nucleus (Vim) MRgFUS underwent DBS along a Vim-posterior subthalamic area (PSA) trajectory. Microelectrode activity was attenuated in the lesioned thalamus; Vim test stimulation was ineffective, whereas PSA stimulation suppressed tremor. A 79-year-old man with recurrent tremor after staged bilateral MRgFUS underwent left DBS targeting the rubral wing.

**Conclusions:**

When the original thalamic target is anatomically or physiologically altered, targeting intact cerebellothalamic pathways provides a rational rescue strategy.

**Highlights:**

Deep brain stimulation can remain effective after failed focused ultrasound thalamotomy. These cases illustrate a lesion-informed, network-based strategy that preserves access to the cerebellothalamic network when prior lesioning has altered local anatomy or physiology.

## Introduction

Magnetic resonance-guided focused ultrasound (MRgFUS) thalamotomy and deep brain stimulation (DBS) are established procedural treatments for medication-refractory essential tremor (ET). MRgFUS provides substantial tremor reduction without an incision, and cohort-level benefit can persist for at least 5 years. Nevertheless, improvement may diminish, and tremor recurrence has been reported in approximately 10% of treated patients [1,2,3]. Early recurrence has been associated with less caudal lesion extension, although recurrence may also reflect lesion evolution or disease progression [3,4].

Management options include repeat lesioning and DBS, but there is no consensus regarding patient selection or the optimal DBS rescue target [4]. Published reports and recent case series show that DBS can be implanted safely within or adjacent to a previous MRgFUS lesion [5,6,7,8]. An unresolved question is how to select a target when lesioning has altered local anatomy or physiology. Because effective tremor stimulation is linked to engagement of a cerebellothalamic network, rescue DBS may benefit from a trajectory that preserves access to network nodes rather than assuming that the previously lesioned region must be either reproduced or avoided [9]. We describe two complementary strategies: a single trajectory spanning the ventral intermediate nucleus (Vim) and posterior subthalamic area (PSA), and direct targeting of the fast gray matter acquisition T1 inversion recovery (FGATIR)-defined rubral wing.

## Case Description

### Case 1

A 71-year-old right-handed man with a 10-year history of medication-refractory ET underwent left Vim MRgFUS for right upper-extremity tremor. Benefit lasted 2 weeks. At DBS assessment 8 months later, his side-specific Fahn-Tolosa-Marin (FTM) score was 30 on the right.

Left DBS was planned using an awake, frame-based, single-lead trajectory spanning the indirectly targeted Vim and the deeper PSA. Microelectrode activity was markedly attenuated from 5 mm above the intended Vim target, corresponding to the prior lesion (Figure 1). Macrostimulation within the expected Vim did not suppress tremor, whereas stimulation below Vim in the PSA produced marked improvement at 2 mA. A directional lead (Vercise Cartesia DB-2202, Boston Scientific) was implanted. Postoperative reconstruction localized the distal contacts below the lesion in the PSA. A normative dentato-rubro-thalamic tract (DRTT) atlas was overlaid, demonstrating overlap between the modeled volume of tissue activated and DRTT fibers below the lesion [10]. At 2 years, the right-sided FTM score was 12, a 60% improvement. Final settings were case positive, contacts 2–4 negative, 3.9 mA, 60 microseconds, and 185 Hz.

**Figure 1:**
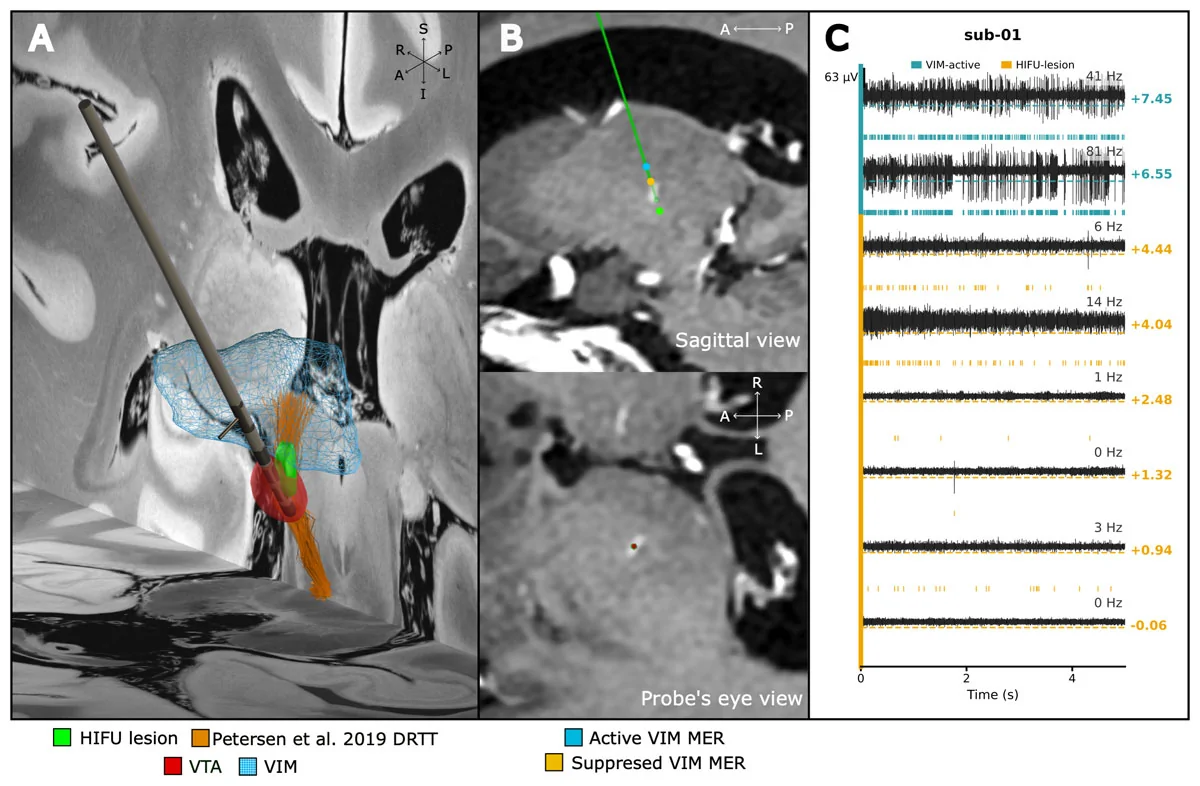
**Case 1. (A)** Postoperative lead reconstruction viewed in an oblique coronal plane. The implanted lead and estimated volume of tissue activated (VTA, red) are shown with the ventral intermediate nucleus (Vim, blue mesh), the prior FUS thalamotomy lesion (green), and the dentato-rubro-thalamic tract (DRTT, orange) reconstructed from a normative tractography atlas. The DRTT is seen traversing the VTA inferior to the lesion, indicating that the electric field engaged cerebellothalamic fibers in the posterior subthalamic area. **(B)** Planned trajectory displayed on the preoperative T1-weighted image in sagittal (top) and probe’s-eye (bottom) views. Colored markers denote microelectrode recording findings along the tract: teal, depths with preserved Vim activity; amber, the depth at which firing became suppressed; green, the final implanted electrode position within the PSA. **(C)** Microelectrode recordings by depth for Case 1. Bandpass-filtered traces (300–3000 Hz, 5 s shown) are stacked from dorsal to ventral, with offline-detected spike events shown as tick marks below each trace and the detection threshold indicated by the dashed line. Depths are color-coded by region: Vim-active (teal, +7.45 and +6.55 mm) and HIFU-lesion (amber, +4.44 mm and below). Firing rate is annotated above each trace and depth relative to the intended target at the right. Spiking activity was suppressed on entering the lesioned territory, consistent with tissue disruption from the prior thalamotomy.

### Case 2

A 79-year-old right-handed man with a 10-year history of medication-refractory ET underwent staged bilateral Vim MRgFUS 6 months apart. Benefit lasted 8 months after the left procedure and 1 week after the right. He developed dysarthria, ataxia, gait imbalance, and dysphagia, which improved only partially. At DBS assessment, his right-sided FTM score was 36.

Left DBS was performed for right-sided tremor. The target was the rubral wing, a hypointense structure visible on FGATIR and proposed imaging correlate of post-decussation DRTT fibers. Intraoperative stimulation produced greater than 80% tremor reduction at 1 mA, and a directional lead (Abbott 6662) was implanted. Lead-DBS reconstruction included manual segmentation of the FGATIR hypointensity and prior MRgFUS lesion; the normative DRTT atlas was displayed for anatomical context (Figure 2) [10]. At 6 months, the right-sided FTM score was 23, a 36% improvement, with clear improvement in drawing spirals. The residual score may partly reflect persistent post-lesional disability, as the functional-disability subscore remained high. Final settings were contact 1 positive, contacts 2, 3, and 4 negative, 4.0 mA, 90 microseconds, and 184 Hz.

**Figure 2:**
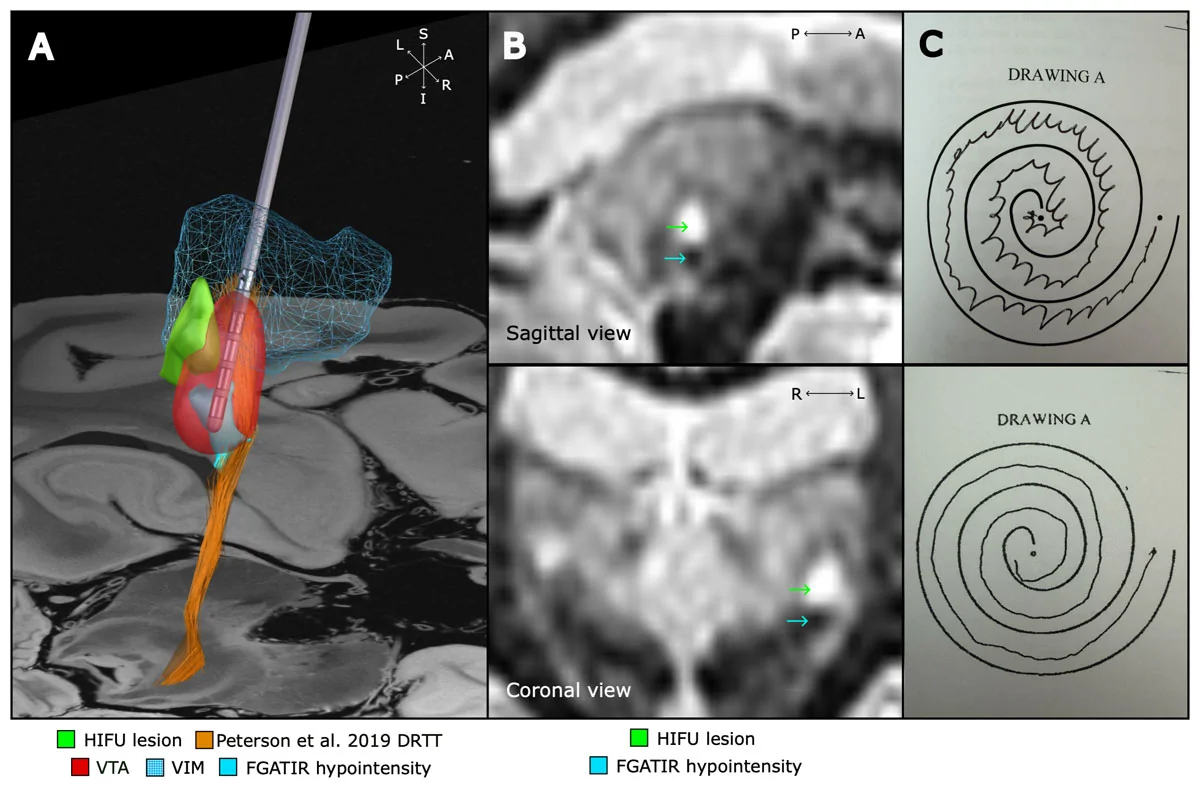
**Case 2**. DBS using direct FGATIR rubral wing targeting following failed FUS thalamotomy. **(A)** Postoperative lead reconstruction. The implanted directional lead and estimated VTA (red) are shown with the Vim (blue mesh), the prior FUS thalamotomy lesion (green), the manually segmented FGATIR hypointensity corresponding to the rubral wing (light blue), and the normative DRTT reconstruction (orange). The prior lesion lies superior and posterior to the FGATIR-defined target, while the VTA overlaps the segmented hypointensity and the DRTT. **(B)** Preoperative FGATIR imaging in sagittal (top) and coronal (bottom) views. Green arrows indicate the prior FUS thalamotomy lesion; cyan arrows indicate the FGATIR hypointensity (rubral wing) used for direct rubral wing targeting. **(C)** Archimedes spiral drawings before (top) and 6 months after (bottom) DBS implantation, demonstrating marked reduction in tremor amplitude.

## Discussion

These cases support DBS as a rescue option after MRgFUS and illustrate one way in which prior lesion anatomy and intraoperative physiology may inform target selection. In Case 1, no FGATIR or tractography was available. Indirect targeting using a Vim-PSA trajectory was therefore performed with intraoperative microelectrode recording (MER) and macrostimulation. Concordance between the lesion and attenuated microelectrode activity, lack of benefit from Vim test stimulation, and immediate benefit at the deeper PSA favored placing therapeutic contacts below the lesion in this patient. This should not be interpreted as evidence that the lesioned Vim was intrinsically unsuitable for DBS or that the lesion must be avoided: successful stimulation within or adjacent to previous MRgFUS lesions has been reported [4,5,6,7,8]. Rather, a Vim-PSA trajectory provides access to multiple stimulation levels when the physiologic boundary of Vim is indistinct, allowing intraoperative testing to identify an effective site [11].

Both strategies are consistent with a network view of tremor therapy. Connectivity analyses have localized effective tremor stimulation to a PSA-centered region extending toward the inferior Vim, supporting modulation of cerebellothalamic pathways across conventional nuclear boundaries [9]. The PSA contains the zona incerta, prelemniscal radiation, and cerebellothalamic fibers, including components of the DRTT [11]. Case 2 used the rubral wing as a directly visible operative landmark. The rubral wing was introduced as an FGATIR correlate of rubrothalamic connections, and subsequent connectomic work localized it to the post-crossing DRTT [12,13]. This provides an anatomical waypoint that does not depend on patient-specific tractography. In the reported cases, DRTT reconstruction was performed exclusively after surgery for anatomical correlation; it was not used for preoperative target selection or intraoperative electrode placement.

Optimal treatment locations for MRgFUS and DBS may overlap without being identical. In a comparative probabilistic mapping study, Sarica et al. found that optimal MRgFUS maps varied with targeting method, including ventral Vim-PSA and dorsal Vim-Vop regions, whereas optimal DBS volumes were confined to the Vim-Vop border [14]. These modality-specific spatial patterns provide an additional rationale for why DBS may succeed after an inadequate or transient response to MRgFUS, including when the DBS target lies within the same general thalamic region or an overlapping network.

Based on these cases, we offer a practical network-based and lesion-informed workflow as one option for rescue DBS. The prior lesion should be reconstructed, and an electrode strategy that preserves access to the cerebellothalamic network can be considered. Intraoperative physiology and stimulation can be used to help guide final placement.

Repeat MRgFUS may remain reasonable after a favorable initial response when a discrete untreated target can be identified, whereas DBS may be preferable for bilateral or progressive symptoms, persistent adverse effects, or a need for adjustability [4]. The principal strengths of this report are multimodal lesion and lead reconstruction, intraoperative physiologic sampling in Case 1, and objective treated-side outcomes. These observations illustrate that a network-level, lesion-informed approach can preserve therapeutic options when the original thalamic site has been altered. More broadly, DBS remains viable after unsuccessful MRgFUS because stimulation can sample and modulate different cerebellothalamic nodes and because the optimal locations for stimulation and lesioning may not be identical.

## Data Availability

The de-identified data supporting this report are available from the corresponding author on reasonable request, subject to institutional and privacy requirements.
